# Fosaprepitant as combination therapy to prevent chemotherapy-induced vomiting in children: a meta-analysis

**DOI:** 10.3389/fphar.2025.1509928

**Published:** 2025-01-23

**Authors:** Yanshuo Shi, Yuanyuan Yue, Yue Zhang, Guoxun Pang

**Affiliations:** ^1^ Department of Pharmacy, Hebei General Hospital, Shijiazhuang, China; ^2^ Hebei Key Laboratory of Clinical Pharmacy, Shijiazhuang, Hebei, China

**Keywords:** fosaprepitant, chemotherapy, pediatric cancer, antiemetic prophylaxis, meta-analysis

## Abstract

**Objective:**

To systematically evaluate the clinical efficacy and safety of fosaprepitant combined with 5-hydroxytryptamine 3 receptor antagonists (5-HT_3_RA) (with or without dexamethasone) on the chemotherapy-induced vomiting in pediatric cancer patients.

**Methods:**

PubMed, Embase, Cochrane Library, China Journal full-text database (CNKI), Wanfang data knowledge service platform (Wanfang) and VIP Chinese sci-tech Journal full-text database (VIP) were searched by computer (retrieval time from database establishment to Apr. 2024), randomized controlled trials (RCTs) and cohort studies about fosaprepitant and 5-HT_3_RA with or without dexamethasone (observation group) versus 5-HT_3_RA, with or without dexamethasone, as the control group for chemotherapy-induced vomiting were collected, after data extraction and quality evaluation, meta-analysis was carried out by Rev Man 5.3 software.

**Results:**

A total of 731 patients were included in 7 trials. Meta-analysis results showed that the complete response (CCR, no vomiting/rescue medication) rates were higher in the observation group compared to that in the control group during the acute [the relative risk: RR = 1.64, 95% confidence interval: 95%CI = 1.35–1.99, P < 0.00001], delayed vomiting [RR = 2.05, 95%CI = 1.32–3.17, P = 0.001] and overall phases [RR = 2.08, 95%CI = 1.69–2.57, P < 0.00001], with statistical significance (P < 0.05). The subgroup analysis of salvage treatment proportion revealed that the need for rescue medication was higher for patients in the control than fosaprepitan regimens [RR = 0.20, 95%CI = 0.08–0.54, P = 0.001] There was no difference in the incidence of adverse drug reaction between two groups [RR = 0.95, 95%CI = 0.75–1.19, P = 0.66].

**Conclusion:**

Fosaprepitant in combination with 5-HT3RA (with or without dexamethasone) has the same safety and more effective in preventing chemotherapy-induced vomiting than 5-HT3RA with or without dexamethasone.

## 1 Introduction

Chemotherapy-induced nausea and vomiting (CINV) is one of the most prevalent and challenging adverse drug reactions encountered in pediatric cancer patients (Herrstedt et al., 2023). Statistics indicate that approximately 70% of children worldwide experience CINV to varying degrees. Beyond diminishing the quality of life for pediatric patients, CINV can significantly affect medication adherence, nutritional status, and electrolyte balance in children (Flank et al., 2017). Consequently, effective prevention of CINV during chemotherapy is crucial for reducing the incidence of vomiting and enhancing the overall quality of life for affected children.

Current guidelines recommend that the primary clinical treatment for CINV involves the use of the neurokinin-1 receptor antagonists (NK-1RA) in combination with 5-HT_3_RA and corticosteroids, such as dexamethasone (Hesketh et al., 2020). Fosaprepitant, a selective NK-1RA, exerts antiemetic effects by competitively inhibiting the binding of neurokinin to NK-1 receptors (Karthaus et al., 2019). Initially, the triple antiemetic prophylactic regimen of fosaprepitant, 5-HT_3_RA, and dexamethasone was primarily utilized in adults, as pediatric patients exhibited unique susceptibility factors that limited its widespread application. However, with the accumulation of clinical experience, there has been a gradual increase in reports regarding the use of fosaprepitant in conjunction with 5-HT_3_RA and dexamethasone to prevent CINV in children under 12 years of age. Despite this growing body of literature, individual reports often feature small sample sizes, and systematic evidence remains lacking. Consequently, medical evidence continues to highlight uncertainties surrounding the efficacy and safety of this combination regimen for preventing CINV in pediatric populations. This aim of this article is to systematically evaluate the efficacy and safety of fosaprepitant in combination with 5-HT_3_RA (with or without dexamethasone) in the prevention of CINV, with the aim of providing evidence-based insights to guide the clinical selection of rational drug regimens.

## 2 Materials and methods

### 2.1 Inclusion and exclusion criteria

#### 2.1.1 Study type

This review includes RCTs and cohort studies, irrespective of language.

#### 2.1.2 Study subjects

The study subjects are children aged 17 years or younger who have been pathologically diagnosed with malignant tumors and are receiving chemotherapy.

#### 2.1.3 Intervention measures

In the experimental group, participants received fosaprepitant in combination with 5-HT_3_RA (with or without dexamethasone). In contrast, children in the control group were treated with 5-HT_3_RA, also with or without dexamethasone. The use of other drugs was consistent across both groups.

#### 2.1.4 Outcome indicators

The primary outcome indicators include: a. The vomiting control rate during the overall observation period; b. The vomiting control rate during the acute CINV stage (the first 24 h following chemotherapy administration); c. The vomiting control rate during the delayed CINV stage (24–120 h post-chemotherapy); d. The proportion of rescue antiemetic drugs administered; e. The incidence of adverse reactions.

#### 2.1.5 Exclusion criteria

Studies were excluded if data could not be extracted or were incomplete. For studies that were published multiple times, the version with the most comprehensive data was selected.

### 2.2 Search strategy

A comprehensive computer search was conducted across several databases, including Embase, PubMed, Cochrane Library, CNKI, Wanfang Data, and VIP, covering the period from the establishment of each database up to April 2024. The search terms utilized were “fosaprepitant”, “5-HT_3_”, “antiemetic prophylaxis”, “pediatric cancer”, “CINV”. The detailed search strategies for each database and the search results are presented in the Supplementary Material S1.

### 2.3 Data extraction and quality assessment

Two researchers independently screened the literature to determine eligibility for inclusion. The extracted data encompassed the title, authors, subjects, methods, measures, outcomes, blinding, and allocation concealment, among other factors. The risk of bias for the included RCTs was assessed using the Cochrane Risk of Bias tool (Linlin et al., 2023). The assessed content included random sequence generation, allocation concealment, blinding of subjects and experimenters, blinding of outcome assessments, incomplete outcome data, selective reporting and other biases. Each study was classified into low risk, unclear, and high risk. Assessment of the methodological quality of cohort studies using the Newcastle-Ottawa Scale (NOS) from three items: selection, comparability, and exposure, Each trail was assigned a score of 0–9. The trials scored ≥7 were considered to be of high quality. Two evaluators independently assessed the quality, with a third individual involved to reach a consensus in cases of disagreement.

### 2.4 Statistical methods

Statistical analysis was conducted using Rev Man 5.3 software. Dichotomous variables were represented by the relative risk (RR) with the corresponding 95% confidence interval (CI), while the continuous variables were expressed as mean difference (MD) with 95% CI. The heterogeneity test, which used to check whether the results of individual studies are unifiable, was analyzed by *Q* test based on Chi square and *I* ^*2*^ statistic. When there was no significant heterogeneity among the studies *(I* ^*2*^ ≤ 50%, *P* ≥ 0.1), a fixed effect model was employed for calulate the pooled RR and MD. Conversely, a random effects model is applied when significant heterogeneity was present (*I* ^*2*^ > 50%, *P* < 0.1) (Sedgwick, 2012). Moreover, sensitivity analyses were conducted to examie the stability of the results by removing each study one by one. The publication bias was investigated using funnel plots.

## 3 Results

### 3.1 Basic information of the included studies

**FIGURE 1:**
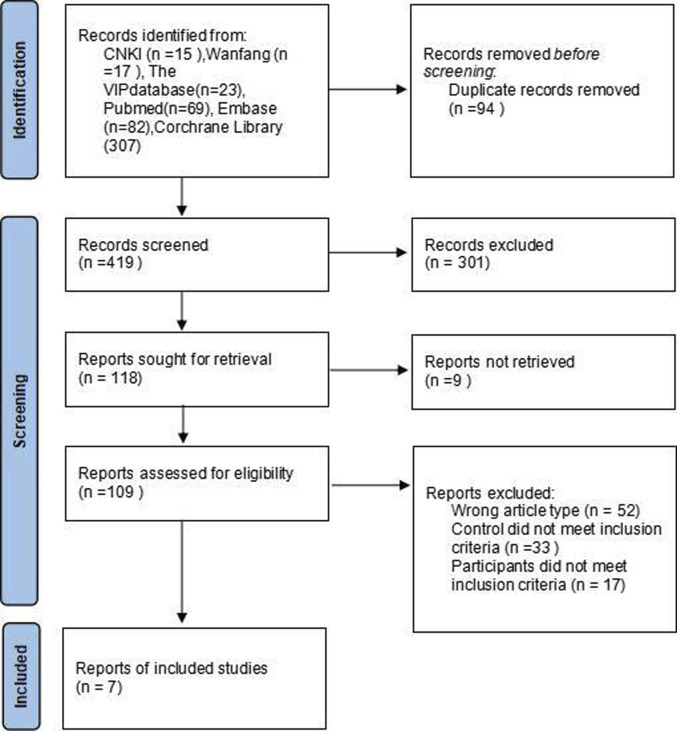
PRISMA flowchart of literature search.

This study included seven literature that met the specified criteria (Mora et al., 2019; Cabanillas Stanchi et al., 2019; Cabanillas Stanchi et al., 2020; Radhakrishnan et al., 2019; Willier et al., 2019; Cabanillas et al., 2020; Liting et al., 2023), comprising 731 cases in total (386 cases in the observation group and 345 cases in the control group). The screening process is illustrated in Figure 1. The sample size in the observation group ranged from 34 to 82 cases. Participants in the observation group received a combination of fosaprepitant, 5-HT_3_ receptor antagonists (5-HT_3_RAs), and dexamethasone. In contrast, the control group was administered only 5-HT_3_RAs and dexamethasone. The basic information regarding the literature is presented in Table 1.

**TABLE 1:** General information of included studies.

First author year of publication	Sample size/case		Male/female/case		Age/year		Age/year		Outcome indicators
	T	C	T	C	T	C	T	C	
Morae et al. (2019)	74	35	42/32	18/17	5.02 ± 3.53	10.36 ± 4.64	Fosaprepitant + Ondansetron + Dexamethasone	Placebo + Ondansetron + Dexamethasone	a, d, e
Cabanillas Stanchi et al. (2019)	41	42	22/19	23/17	3.0 ± 0.9	2.9 ± 0.8	Fosaprepitant + Granisetron ± Dexamethasone	Granisetron ± Dexamethasone	a, b, c, d, e
Cabanillas Stanchi et al. (2020)	35	35	26/9	21/14	4.8	5.1	Fosaprepitant + Ondansetron/Granisetron	Ondansetron/Granisetron	a, b, c, d, e
Radhakrishnan et al. (2019)	81	82	48/33	51/31	6	5	Fosaprepitant + Ondansetron + Dexamethasone	Placebo + Ondansetron + Dexamethasone	a, b, c, d, e
Willier et al. (2019)	40	39	25/15	22/17	7.4	8.3	Fosaprepitant + ondansetron	Ondansetron	a, b, c, e
Cabanillas Stanchi et al. (2020)	60	60	35/25	32/28	11.7	11.8	Fosaprepitant + Granisetron	granisetron	a, b, c, e
Liting et al. (2023)	55	52	30/25	21/31	3.3	3.8	Fosaprepitant + ondansetron + dexamethasone	Ondansetron + Dexamethasone	a, b, c, e

The study involved two groups: the observation group (T) and the control group (C). The following metrics were evaluated: a. Total vomiting control rate; b. Acute phase vomiting control rate; c. Delayed phase vomiting control rate; d. Emergency antiemetic rate; e. Incidence rate of adverse reactions.

### 3.2 Evaluation of methodological quality and risk assessment of included studies

**FIGURE 2:**
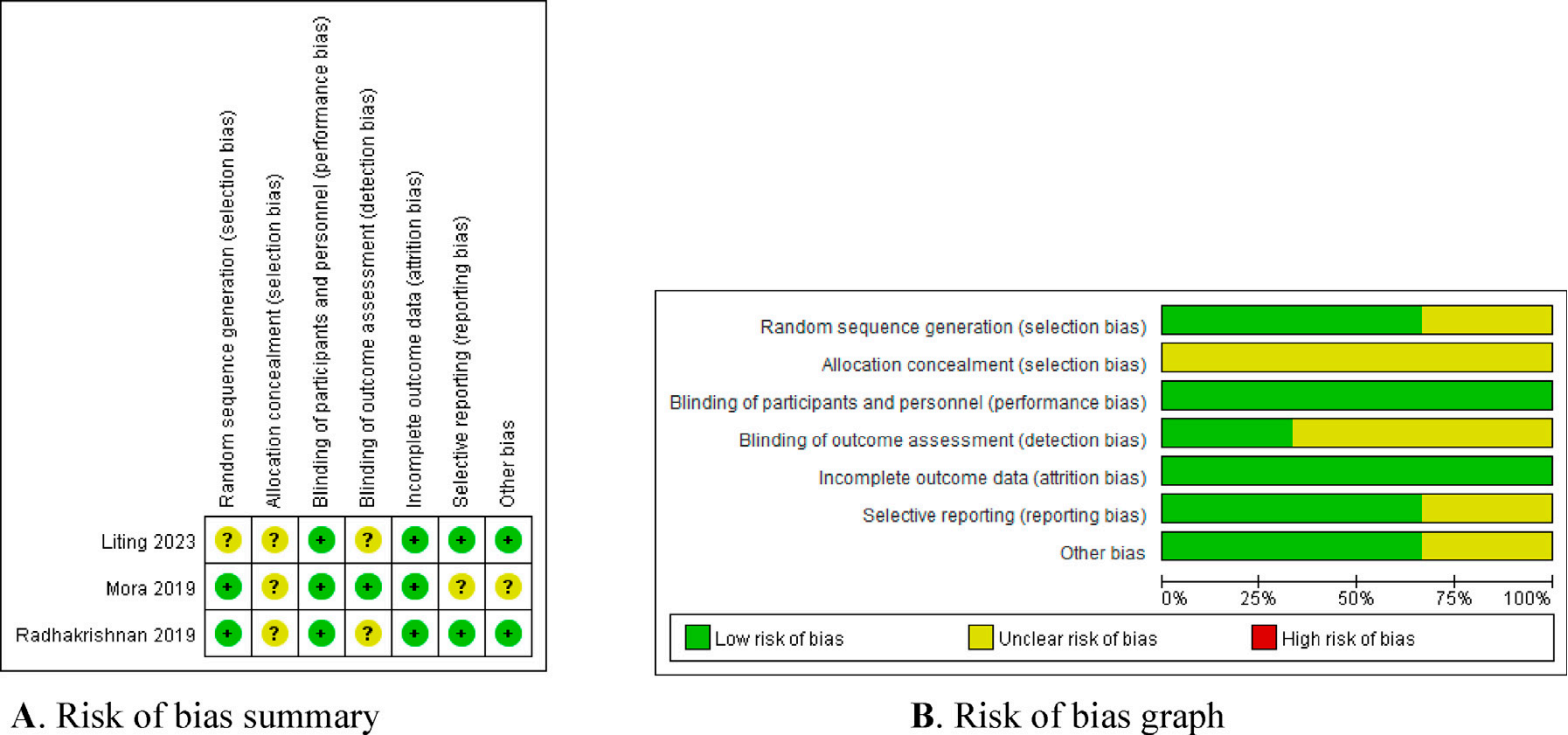
Bias risk assessment of included literature. **(A)** Risk of bias summary. **(B)** Risk of bias graph.

Four of the seven included studies were cohort controlled and three were randomised controlled. The three randomized trials identified were assessed by using the Cochrane risk of bias tool. The study by Liling et al. did not describe the random allocation method or whether allocation concealment was implemented, and its blinding method was insufficient. The study by Mora et al. did not specify whether allocation concealment was used, and it is unclear if there is any selective publication bias or other biases present. The study by Radhakrishnan et al. (2019) did not describe whether allocation concealment was implemented, and its blinding method was also insufficient. The results of the risk of bias assessment are presented in Figure 2. The quality of cohort studies was assessed using the Newcastle-Ottawa Scale (NOS), and the results are shown in Table 2.

**TABLE 2:** The quality assessment according to Newcastle-Ottawa Scale.

Author	Representativenss of the exposed cohort	Selection of the nonexposed cohort	Ascertalnment of exposure	Demonstration that outcome of interest was not present at start of study	Comparability of cohorts on the basis of the design or analysis	Assessment of outcome	Was follow up long enough for outcomes to occur	Adequacy of follow up of cohorts	Total scores
Cabanillas Stanchi et al., 2019 ^[9]^	★	★	★	★	★★	★	★		8
Cabanillas Stanchi et al., 2020 ^[10]^	★	★	★	★	★★		★	★	8
Willier et al., 2019 ^[12]^	★	★	★	★	★★		★	★	8
Cabanillas et al., 2020 ^[13]^	★	★	★	★	★★	★	★	★	9

### 3.3 Meta-analysis results

#### 3.3.1 Overall control rate of vomiting

**FIGURE 3:**
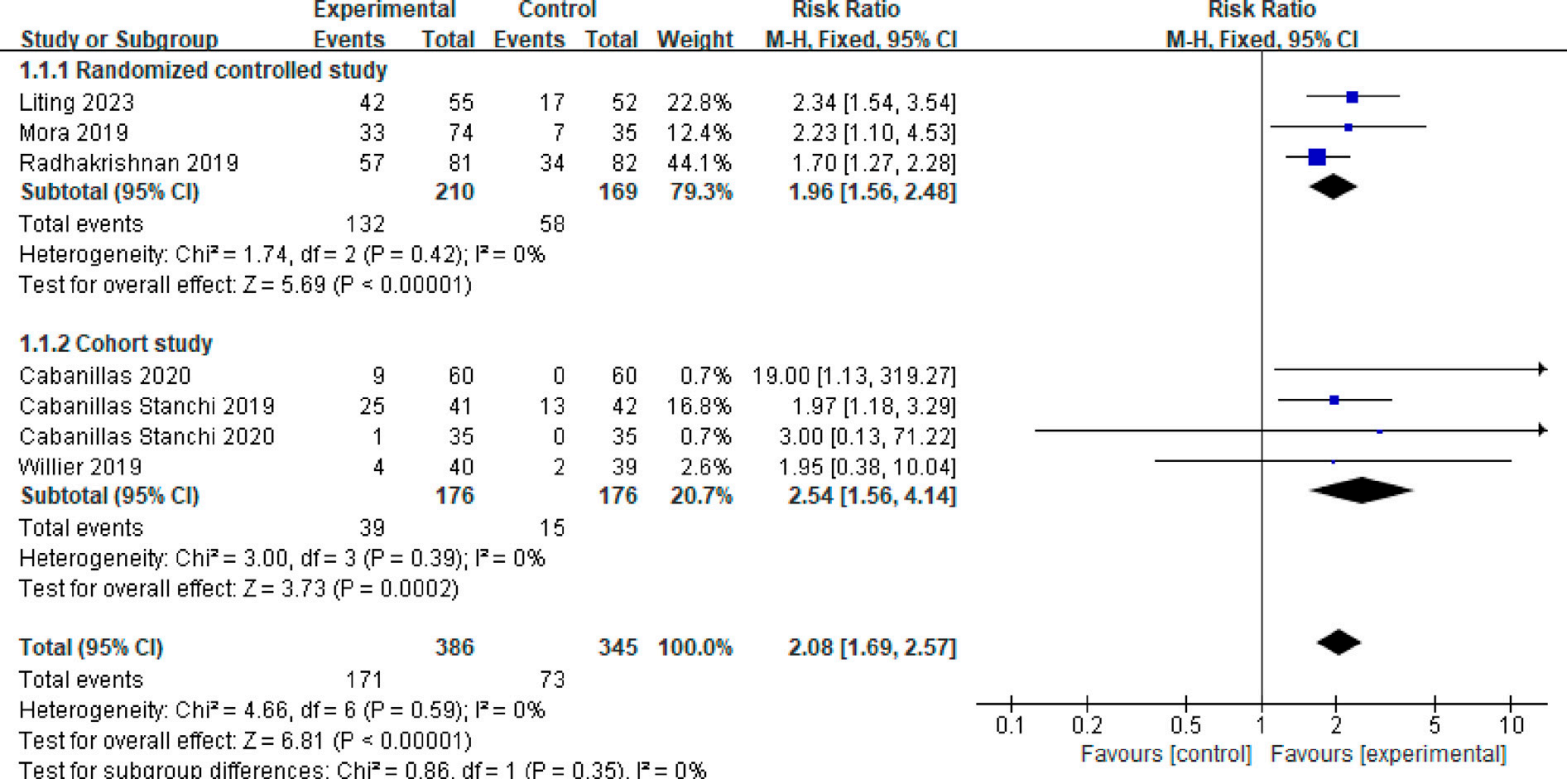
Meta-analysis of total control rate of vomiting in two groups.

Seven studies reported the overall control rate of vomiting (Mora et al., 2019; Cabanillas Stanchi et al., 2019; Cabanillas Stanchi et al., 2020; Radhakrishnan et al., 2019; Willier et al., 2019; Cabanillas et al., 2020; Liting et al., 2023). A fixed effects model was employed after testing for heterogeneity (*P* = 0.59, *I* ^*2*^ = 0%). The combined effect size was calculated as RR = 2.08 (95%CI = 1.69–2.57, *P* < 0.00001), indicating a statistically significant difference. Subgroup analysis was conducted based on the type of study. Among the studies, three were randomized controlled trials (Mora et al., 2019; Radhakrishnan et al., 2019; Liting et al., 2023). The meta-analysis results revealed no heterogeneity between these studies (*P* = 0.42, *I* ^*2*^ = 0%). In the observation group, the total vomiting control rate was significantly higher than that of the control group (RR = 1.96, 95%CI = 1.56–2.48, *P* < 0.00001). Moreover, four studies were identified as cohort studies (Cabanillas Stanchiet al. 2019; Cabanillas Stanchiet et al., 2020; Willier et al., 2019; Cabanillas et al., 2020). The meta-analysis results indicated no heterogeneity among these studies (*P* = 0.39, *I* ^*2*^ = 0%). The overall vomiting control rate in the observation group was also significantly higher compared to the control group (RR = 2.54, 95%CI = 1.56–4.14, *P* = 0.0002). Refer to Figure 3 for a visual representation.

#### 3.3.2 Vomiting control rate in the acute phase

**FIGURE 4:**
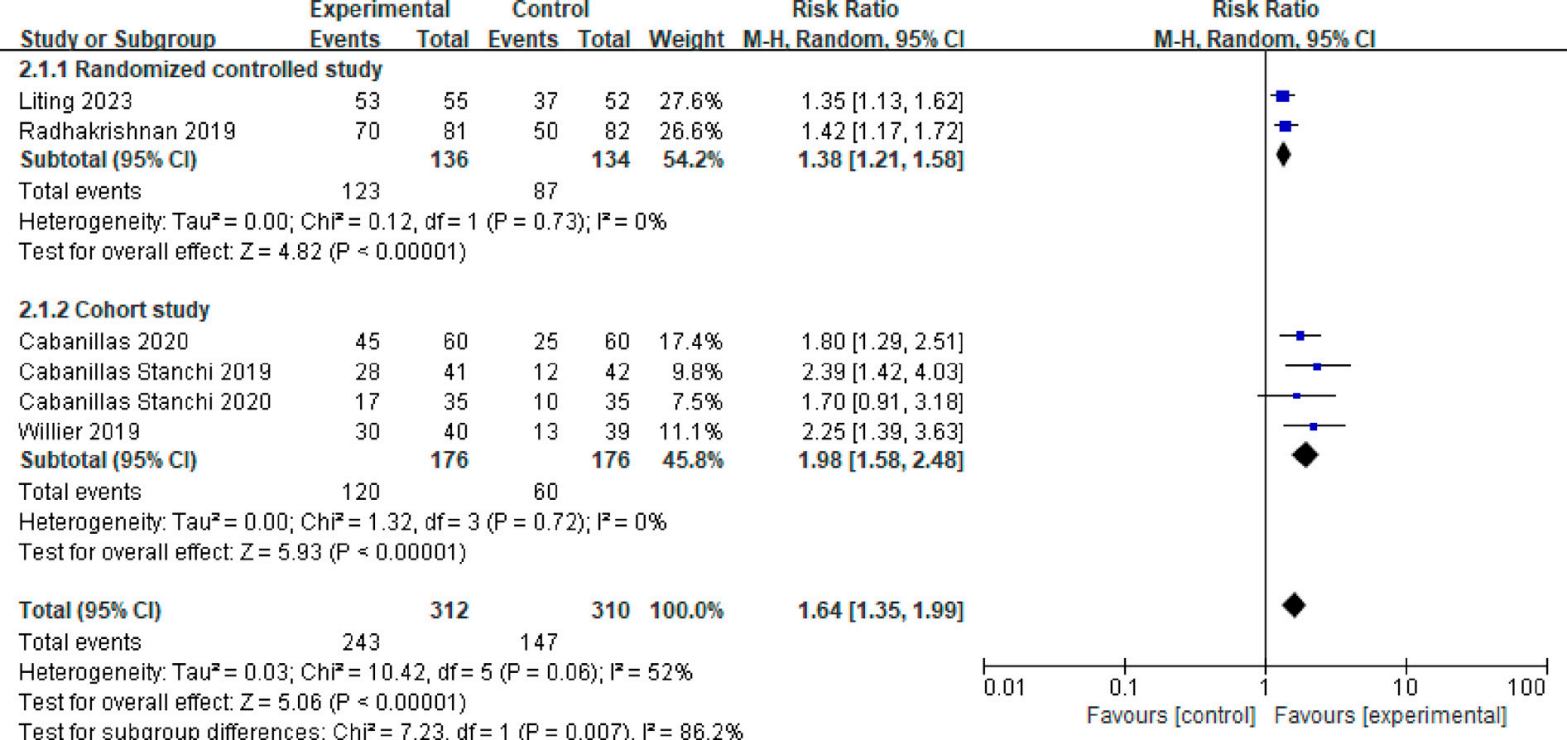
Meta-analysis of the control rate of acute vomiting in two groups.

Six studies reported the vomiting control rate during the acute phase (Cabanillas Stanchi et al., 2019; Cabanillas Stanchi et al., 2020; Radhakrishnan et al., 2019; Willier et al., 2019; Cabanillas et al., 2020; Liting et al., 2023). After testing for heterogeneity (*P* = 0.06, *I* ^*2*^ = 52%), a random effects model was employed, yielding a combined effect size of RR = 1.64 (95%CI = 1.35–1.99, *P* < 0.00001), indicating a statistically significant difference. A subgroup analysis was performed based on study type. Two studies were randomized controlled trials (Radhakrishnan et al., 2019; Liting et al., 2023), and the meta-analysis results revealed no heterogeneity among these studies (*P* = 0.73, *I* ^*2*^ = 0%). The results indicated that the vomiting control rate in the acute phase was significantly higher in the observation group compared to the control group, with a statistically significant difference (RR = 1.38, 95%CI = 1.21–1.58, *P* < 0.00001). Four studies were cohort studies (Cabanillas Stanchi et al., 2019; Cabanillas Stanchi et al., 2020; Willier et al., 2019; Cabanillas et al., 2020), and the meta-analysis results showed no heterogeneity among these studies (*P* = 0.72*, I* ^*2*^ = 0%). The control rate of vomiting in the acute phase for the observation group was significantly higher than that of the control group, with a statistically significant difference (RR = 1.98, 95%CI = 1.58–2.48, *P* < 0.00001). See Figure 4 for more information.

#### 3.3.3 Control rate of vomiting in the delayed phase

**FIGURE 5:**
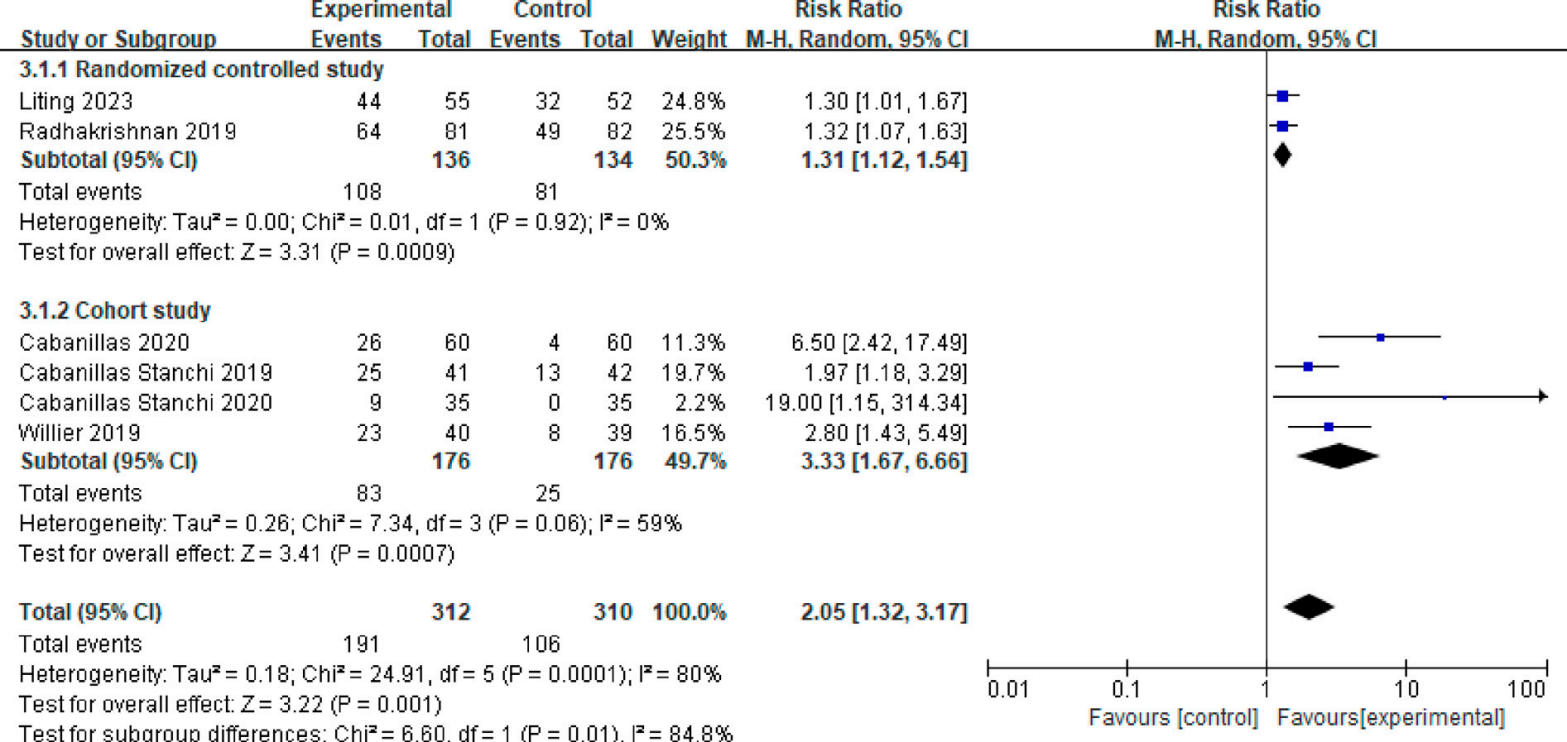
Meta-analysis of the control rate of delay vomiting in two groups.

Six studies reported on the control rate of vomiting in the delayed phase (Cabanillas Stanchi et al., 2019; Cabanillas Stanchi et al., 2020; Radhakrishnan et al., 2019; Willier et al., 2019; Cabanillas et al., 2020; Liting et al., 2023). Following a heterogeneity test (*P* < 0.001, *I* ^*2*^ = 80%), a random effects model was employed, resulting in a combined effect size of RR = 2.05 (95%CI = 1.32–3.17, *P* = 0.001), indicating a statistically significant difference. A subgroup analysis was performed based on different study types. Among these, two studies were identified as randomized controlled trials (Radhakrishnan et al., 2019; Liting et al., 2023). The meta-analysis results revealed no heterogeneity between these studies (*P* = 0.92, *I* ^*2*^ = 0%). The findings indicated that the vomiting control rate in the delayed phase for the observation group was significantly higher than that of the control group, with a statistically significant difference (RR = 1.31, 95%CI = 1.12–1.54, *P* = 0.0009). Additionally, four studies were categorized as randomized controlled trials (Cabanillas Stanchi et al., 2019; Cabanillas Stanchi et al., 2020; Willier et al., 2019; Cabanillas et al., 2020). The meta-analysis results for this group showed moderate heterogeneity (*P* = 0.06, *I* ^*2*^ = 59%). The control rate of delayed vomiting in children from the observation group was significantly higher than that of the control group, with a statistically significant difference (RR = 3.33, 95%CI = 1.67–6.066, *P* = 0.0007), as illustrated in Figure 5.

#### 3.3.4 Emergency antiemetic ratio

**FIGURE 6:**
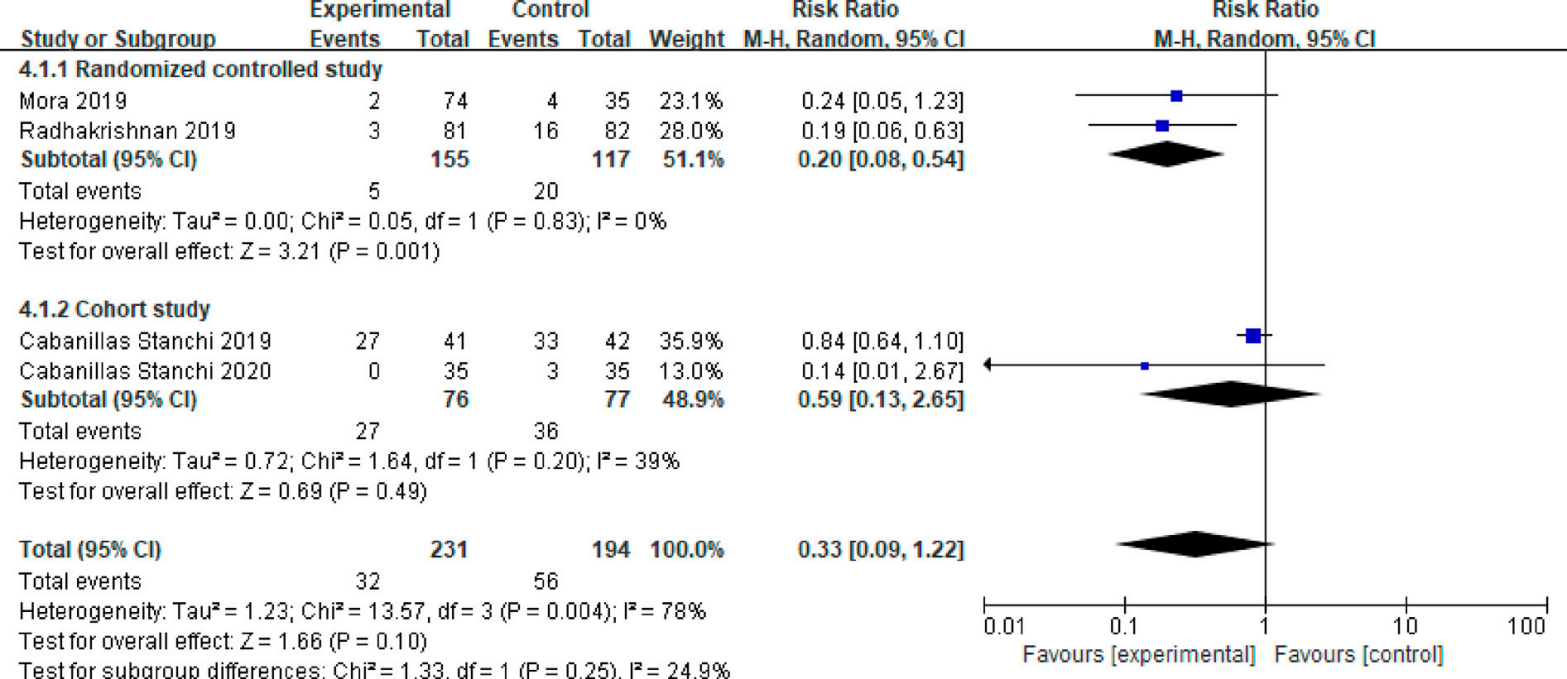
Meta-analysis of emergency antiemetic rate in two groups.

Four studies reported the emergency antiemetic patient ratio (Mora et al., 2019; Cabanillas Stanchi et al., 2019; Cabanillas Stanchi et al., 2020; Radhakrishnan et al., 2019). After testing for heterogeneity (*P* = 0.004, *I* ^*2*^ = 78%), a random effects model was employed, resulting in a combined effect size of (RR = 0.33, 95%CI = 0.09–1.22, *P* = 0.10). This difference was not statistically significant. A subgroup analysis was conducted based on different study types. Two of the studies were randomized controlled trials (Mora et al., 2019; Radhakrishnan et al., 2019). The meta-analysis results indicated no heterogeneity between these studies (*P* = 0.83, *I* ^*2*^ = 0%). In this subgroup, the emergency response rate of children in the observation group demonstrated a lower usage rate of antiemetic drugs compared to the control group, with a statistically significant difference (RR = 0.20, 95%CI = 0.08–0.54, *P* = 0.001). The other two studies were cohort studies (Cabanillas Stanchi et al., 2019; Cabanillas Stanchi et al., 2020), and the meta-analysis results revealed slight heterogeneity between these studies (*P* = 0.20, *I* ^*2*^ = 39%). In this subgroup, there was no statistically significant difference in the use rate of emergency antiemetic drugs between the two groups of children (RR = 0.59, 95%CI = 0.13–2.65, *P* = 0.49). Details in Figure 6.

#### 3.3.5 Incidence of adverse reactions

**FIGURE 7:**
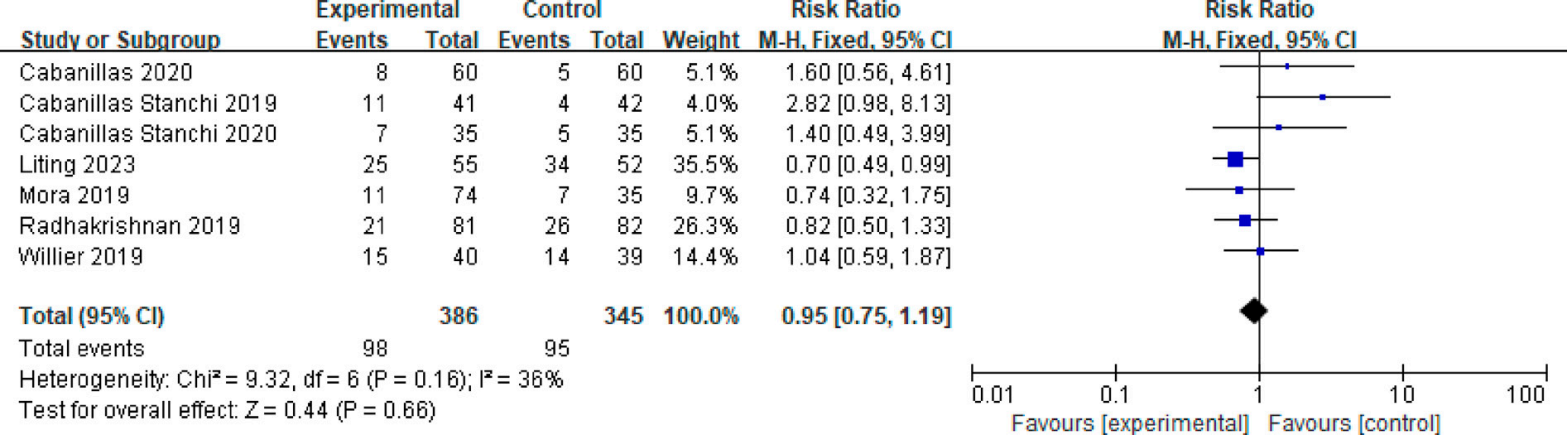
Meta-analysis of incidence of adverse reactions in two groups.

Seven studies reported the incidence of adverse reactions (Mora et al., 2019; Cabanillas Stanchi et al., 2019; Cabanillas Stanchi et al., 2020; Radhakrishnan et al., 2019; Willier et al., 2019; Cabanillas et al., 2020; Liting et al., 2023). A fixed effects model was employed after assessing heterogeneity (*P* = 0.16, *I* ^*2*^ = 36%). The meta-analysis results indicate that the difference in the incidence of adverse reactions between children in the observation group and those in the control group is minimal, and the results are not statistically significant (RR = 0.95, 95%CI = 0.75–1.19, *P* = 0.66), as illustrated in Figure 7.

### 3.4 Sensitivity analysis

Sensitivity analysis was performed by sequentially excluding each included study. The results are presented in Table 3. As indicated in the table, after the exclusion of the study by Cabanillas Stanchi et al., 2019, the *I* ^*2*^ of the emergency antiemetic rate decreased from 78% to 0%, and the effect size exhibited a directional change (*P* = 0.0005). Among the four studies that reported on emergency antiemetic outcomes (Morae et al., 2019; Cabanillas Stanchi et al., 2019; Cabanillas Stanchi et al., 2020; Radhakrishnan et al., 2019), the combination of methodological factors, such as study type and intervention measures in the study by Karin, may account for the observed high heterogeneity. The results for other outcome indicators remained relatively unchanged following the sequential exclusion of studies, with no statistically significant differences noted. These findings suggest that the results of this meta-analysis are both stable and highly credible.

**TABLE 3:** The result of sensitivity analysis.

Outcome indicator	Exclusion of literature	Heterogeneity	RR (*95%*CI)	*P*
Total vomiting control rate	Unexcepted	*P* = 0.59, *I* ^*2*^ = 0%	2.08 (1.69,2.57)	*P* < 0.00001
	Morae et al. (2019)	*P* = 0.48, *I* ^*2*^ = 0%	2.06 (1.66,2.57)	*P* < 0.00001
	Cabanillas Stanchi et al. (2019)	*P* = 0.45, *I* ^*2*^ = 0%	2.11 (1.67,2.65)	*P <* 0.00001
	Cabanillas Stanchi et al. (2020)	*P* = 0.47, *I* ^*2*^ = 0%	2.08 (1.68,2.57)	*P* < 0.00001
	Radhakrishnan et al. (2019)	*P* = 0.74, *I* ^*2*^ = 0%	2.39 (1.78,3.20)	*P* < 0.00001
	Willier et al. (2019)	*P* = 0.46, *I* ^*2*^ = 0%	2.09 (1.69,2.58)	*P* < 0.00001
	Cabanillas Stanchi et al. (2020)	*P* = 0.87, *I* ^*2*^ = 0%	1.97 (1.60,2.43)	*P* < 0.00001
	Liting et al. (2023)	*P* = 0.57, *I* ^*2*^ = 0%	2.01 (1.57,2.56)	*P* < 0.00001
Acute phase vomiting control rate	Unexcepted	*P* = 0.09, *I* ^*2*^ = 47%	1.63 (1.43,1.84)	*P* < 0.00001
	Cabanillas Stanchi et al. (2019)	*P* = 0.16, *I* ^*2*^ = 39%	1.57 (1.38,1.84)	*P* < 0.00001
	Cabanillas Stanchi et al. (2020)	*P* = 0.05, *I* ^*2*^ = 57%	1.63 (1.33,1.99)	*P* < 0.00001
	Radhakrishnan et al. (2019)	*P* = 0.06, *I* ^*2*^ = 57%	1.75 (1.35,2.27)	*P* < 0.0001
	Willier et al. (2019)	*P* = 0.19, *I* ^*2*^ = 34%	1.57 (1.38,1.78)	*P* < 0.00001
	Cabanillas Stanchi et al. (2020)	*P* = 0.09, *I* ^*2*^ = 51%	1.59 (1.30,1.96)	*P* < 0.0001
	Liting et al. (2023)	*P* = 0.19, *I* ^*2*^ = 34%	1.72 (1.47,2.01)	*P* < 0.00001
Delayed phase vomiting control rate	Unexcepted	*P* < 0.0001, *I* ^*2*^ = 83%	2.01 (1.21,3.33)	*P* = 0.007
	Cabanillas Stanchi et al. (2019)	*P* < 0.0001, *I* ^*2*^ = 86%	2.09 (1.13,3.84)	*P* = 0.02
	Cabanillas Stanchi et al. (2020)	*P* < 0.0001, *I* ^*2*^ = 84%	1.85 (1.15,2.97)	*P* = 0.01
	Radhakrishnan et al. (2019)	*P* < 0.000 01, *I* ^*2*^ = 87%	2.60 (1.14,5.59)	*P* = 0.02
	Willier et al. (2019)	*P* < 0.0001, *I* ^*2*^ = 84%	1.86 (1.08,3.18)	*P* = 0.02
	Cabanillas Stanchi et al. (2020)	*P* = 0.002, *I* ^*2*^ = 76%	1.60 (1.05,2.44)	*P* = 0.03
	Liting et al. (2023)	*P* < 0.0001, *I* ^*2*^ = 83%	2.66 (1.33,5.33)	*P* = 0.006
Emergency antiemetic rate	Unexcepted	*P* = 0.004, *I* ^*2*^ = 78%	0.33 (0.09,0.22)	*P* = 0.10
	Morae et al. (2019)	*P* = 0.005, *I* ^*2*^ = 81%	0.35 (0.07,1.75)	*P* = 0.20
	Cabanillas Stanchi et al. (2019)	*P* = 0.95, *I* ^*2*^ = 0%	0.19 (0.08,0.49)	*P* = 0.0005
	Cabanillas Stanchi et al. (2020)	*P* = 0.004, *I* ^*2*^ = 82%	0.37 (0.09,1.49)	*P* = 0.16
	Radhakrishnan et al. (2019)	*P* = 0.11, *I* ^*2*^ = 55%	0.46 (0.13,1.58)	*P* = 0.21
Incidence of adverse reactions	Unexcepted	*P* = 0.16, *I* ^*2*^ = 36%	0.95 (0.75,1.19)	*P* = 0.66
	Morae et al. (2019)	*P* = 0.10, *I* ^*2*^ = 46%	0.97 (0.77,1.23)	*P* = 0.81
	Cabanillas Stanchi et al. (2019)	*P* = 0.52, *I* ^*2*^ = 0%	0.87 (0.69,1.10)	*P* = 0.25
	Cabanillas Stanchi et al. (2020)	*P* = 0.13, *I* ^*2*^ = 41%	0.93 (0.73,1.17)	*P* = 0.52
	Radhakrishnan et al. (2019)	*P* = 0.09, *I* ^*2*^ = 47%	1.00 (0.77,1.29)	*P* = 0.98
	Willier et al. (2019)	*P* = 0.11, *I* ^*2*^ = 44%	0.93 (0.73,1.20)	*P* = 0.59
	Cabanillas Stanchi et al. (2020)	*P* = 0.16, *I* ^*2*^ = 37%	0.91 (0.72,1.16)	*P* = 0.46
	Liting et al. (2023)	*P* = 0.31, *I* ^*2*^ = 16%	1.09 (0.81,1.46)	*P* = 0.46

### 3.5 Publication bias assessment

**FIGURE 8:**
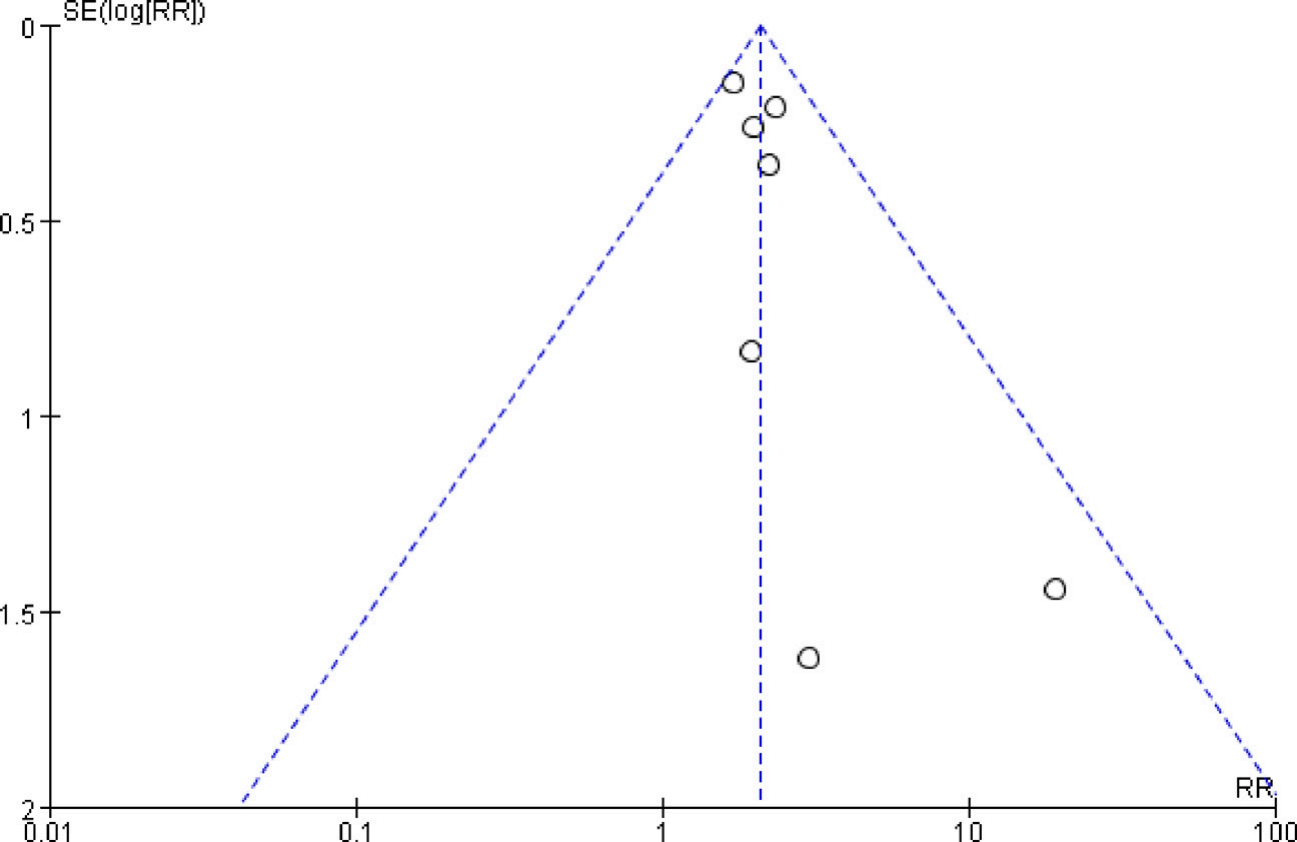
Funnel plot of total control rate of vomiting in two groups.

An analysis of publication bias was conducted on the overall vomiting control rate. The results indicated that the seven included studies were unevenly distributed on both sides of the funnel plot, suggesting the presence of publication bias in this study (Figure 8).

## 4 Discussion

If nausea and vomiting induced by chemotherapy drugs can be effectively managed, the quality of life, treatment adherence, and nutritional status of cancer patients will significantly improve. Current evidence suggests that the onset and progression of CINV are associated with the activation of various neurotransmitter receptors found in the chemosensory trigger zone, the vomiting center, and the gastrointestinal tract. The primary neurotransmitter receptors implicated in the vomiting response include 5-HT_3_, NK-1, and corticosteroids, in addition to acetylcholine, dopamine, and opiates. Chemotherapy agents directly stimulate the chemoreceptor trigger zone, generating nerve impulse signals that lead to central vomiting. Furthermore, these agents activate enterochromaffin cells in the small intestine, which release 5-HT_3_. This neurotransmitter then activates receptors on the vagus nerve and visceral afferent fibers, transmitting signals to the vomiting center and resulting in peripheral vomiting (Navari and Aapro, 2016; Aapro, 2018). Substance P/NK-1 receptor antagonist (NK-1RA) is a novel antiemetic drug that has been developed in recent years. It is also recognized as one of the classic combination drugs recommended by both domestic and international guidelines for the management of CINV (Hospital Pharmacy Professional Committee of the Chinese Pharmaceutical Association, 2022; Herrstedt et al., 2023). Currently, the NK-1 receptor antagonists available in China include aprepitant and fosaprepitant. Fosaprepitant serves as a prodrug of aprepitant, converting into aprepitant within the body to exert its antiemetic effects. Due to the absence of relevant domestic guidelines advocating the use of fosaprepitant in pediatric populations, this article systematically evaluates the efficacy and safety of fosaprepitant as a combination for the prevention of CINV in children with cancer. Except for the study by Radhakrishnan et al. (2019), where the observation group used a Fosaprepitant dose of 3 mg/kg (maximum dose of 150 mg), all other studies used a dose of 4 mg/kg (maximum dose of 150 mg). The meta-analysis results showed a significant increase in the rate of complete control of total, acute phase and delayed phase vomiting with the addition of fosaprepitant compared to 5-HT_3_ receptor antagonist alone (with or without dexamethasone). Regarding the incidence of emergency vomiting events, the combined results of two randomized controlled studies demonstrated that the fosaprepitant group had a significantly lower incidence compared to the control group. Additionally, the combined results of two cohort studies revealed no statistically significant difference in incidence between the two groups. After conducting subgroup analysis according to the methodology, the heterogeneity among the studies was reduced, indicating that the research methods were one of the reasons for the heterogeneity. Adverse reactions were predominantly mild, including rash, fever, headache and constipation, with no significant differences observed between the two groups and no serious adverse reactions reported. Using funnel plots to assess publication bias for the overall complete control rate of vomiting, the results indicated that the funnel plot was asymmetric. However, due to the limited number of included studies, it was not possible to definitively determine whether publication bias exists.

## 5 Conclusion

For pediatric patients with tumors, younger age makes oral medication more challenging, and chemotherapy-induced nausea and vomiting, along with oral-related adverse effects (Cheng et al., 2008), contribute to poor adherence among these patients. Fosaprepitant, as an intravenous formulation, can enhance patient adherence and ensure the smooth progression of chemotherapy. Additionally, the inclusion of Fosaprepitant can reduce the incidence of vomiting caused by moderate to highly emetogenic chemotherapy and decrease the usage of rescue antiemetic medications, demonstrating good tolerability in patients. However, this study has the following limitations: 1) The number of included studies is small. It is well known that large sample randomized controlled trials (RCTs) are the primary evidence in evidence-based medicine and have higher reliability; however, this study only included three RCTs. 2) The sample sizes in the literature are small and uneven. 3) The intervention measures varied across studies, including the selection of 5-HT3 receptor antagonists and rescue antiemetics, as well as the use of dexamethasone alone or in combination, which may be a primary reason for the heterogeneity observed. These factors may contribute to the asymmetry of the funnel plot. Therefore, further multicenter, prospective randomized controlled trials are needed to validate these conclusions and provide robust evidence for clinical practice.

## Data Availability

The original contributions presented in the study are included in the article/Supplementary Material, further inquiries can be directed to the corresponding author.

## References

[B1] Aapro M. (2018). CINV: still troubling patients after all these years. Support Care Cancer 26 (Suppl. 1), 5–9. 10.1007/s00520-018-4131-3 29556808 PMC5876280

[B2] Cabanillas Stanchi K. M. Ebinger M. Hartmann U. Queudeville M. Feucht J. Ost M. (2019). Efficacy, safety and feasibility of antiemetic prophylaxis with fosaprepitant, granisetron and dexamethasone in pediatric patients with hemato-oncological malignancies. Drug Des. Devel Ther. 13, 3439–3451. 10.2147/DDDT.S214264 PMC6777642 31686784

[B3] Cabanillas Stanchi K. M. Vek J. Schlegel P. Rupprecht J. V. Flaadt T. Weber S. (2020). Antiemetic prophylaxis with fosaprepitant and granisetron in pediatric patients undergoing allogeneic hematopoietic stem cell transplantation. J. Cancer Res. Clin. 146 (4), 1089–1100. 10.1007/s00432-020-03143-8 PMC7085480 32056007

[B4] Cabanillas Stanchi K. M. Willier S. Vek J. Schlegel P. Queudeville M. Rieflin N. (2020). Antiemetic prophylaxis with fosaprepitant and 5-HT3-receptor antagonists in pediatric patients undergoing autologous hematopoietic stem cell transplantation. Drug Des. Devel Ther. 14, 3915–3927. 10.2147/DDDT.S260887 PMC7524181 33061297

[B5] Cheng K. K. Goggins W. B. Lee V. W. Thompson D. R. (2008). Risk factors for oral mucositis in children undergoing chemotherapy: a matched case-control study. Oral Oncol. 44 (11), 1019–1025. 10.1016/j.oraloncology.2008.01.003 18329325

[B6] Flank J. Sparavalo J. Vol H. Hagen L. Stuhler R. Chong D. (2017). The burden of chemotherapy-induced nausea and vomiting in children receiving hematopoietic stem cell transplantation conditioning: a prospective study. Bone marrow Transplant. 52 (9), 1294–1299. 10.1038/bmt.2017.112 28581463

[B7] Herrstedt J. Celio L. Hesketh P. J. Zhang L. Navari R. Chan A. (2023). 2023 updated MASCC/ESMO consensus recommendations: prevention of nausea and vomiting following high-emetic-risk antineoplastic agents. Support. Care Cancer 32 (1), 47. 10.1007/s00520-023-08221-4 38127246 PMC10739516

[B8] Herrstedt J. Clark-Snow R. Ruhlmann C. H. Molassiotis A. Olver I. Rapoport B. (2023). 2023 MASCC and ESMO guideline update for the prevention of chemotherapy- and radiotherapy-induced nausea and vomiting. ESMO Open 9 (2), 102195. 10.1016/j.esmoop.2023.102195 PMC10937211 38458657

[B9] Hesketh P. J. Kris M. G. Basch E. Bohlke K. Barbour S. Y. Clark-Snow R. A. (2020). Antiemetics: ASCO guideline update. J. Clin. Oncol. 38 (24), 2782–2797. 10.1200/JCO.20.01296 32658626

[B10] Hospital Pharmacy Professional Committee of the Chinese Pharmaceutical Association (2022). Guidelines for the prevention and treatment of chemotherapy-induced nausea and vomiting 42(5), 457–473.

[B11] Karthaus M. Schiel X. Ruhlmann C. H. Celio L. (2019). Neurokinin-1 receptor antagonists: review of their role for the Prevention of chemotherapy-induced nausea and vomiting in adults. Expert Rev. Clin. Pharmacol. 12 (7), 661–680. 10.1080/17512433.2019.1621162 31194593

[B12] Linlin Z. Ziyu Y. Hongyu D. Zhang Y. Liao X. Clarke M. (2023). Citation of updated and co-published Cochrane methodology reviews. Syst. Rev. 12 (1), 120. 10.1186/s13643-023-02270-w 37443094 PMC10347811

[B13] Liting Y. Xingwei S. Zhuo W. Shunguo Z. Yijin G. (2023). Evaluation of the effectiveness and safety of fosaprepitant in preventing nausea and vomiting associated with highly emetogenic chemotherapy drugs in pediatric cancer patients. J. Clin. Pediatr. 41 (8), 604–609. 10.12372/jcp.2023.22e0483

[B14] Mora J. Valero M. DiCristina C. Jin M. Chain A. Bickham K. (2019). Pharmacokinetics/pharmacodynamics, safety, and tolerability of fosaprepitant for the prevention of chemotherapy-induced nausea and vomiting in pediatric cancer patients. Pediatr. Blood and Cancer 66 (6), e27690. 10.1002/pbc.27690 30900392

[B15] Navari R. M. Aapro M. (2016). Antiemetic prophylaxis for chemotherapy induced nausea and vomiting. N. Engl. J. Med. 374 (14), 1356–1367. 10.1056/NEJMra1515442 27050207

[B16] Radhakrishnan V. Joshi A. Ramamoorthy J. Rajaraman S. Ganesan P. Ganesan T. S. (2019). Intravenous fosaprepitant for the prevention of chemotherapy-induced vomiting in children: a double-blind, placebo-controlled, phase III randomized trial. Pediatr. Blood and Cancer 66 (6), e27551. 10.1002/pbc.27551 30426714

[B17] Sedgwick P. (2012). Meta-analyses: tests of heterogeneity. BMJ Br. Med. J. 344 (jun13 2), e3971. 10.1136/bmj.e3971

[B18] Willier S. Cabanillas Stanchi K. M. von H. M. Binder V. Blaeschke F. Feucht J. (2019). Efficacy, safety and feasibility of fosaprepitant for the prevention of chemotherapy-induced nausea and vomiting in pediatric patients receiving moderately and highly emetogenic chemotherapy-results of a non-interventional observation study. BMC Cancer 19 (1), 1118. 10.1186/s12885-019-6252-6 31730451 PMC6858739

