# Supplementary material for: Fosaprepitant as combination therapy to prevent chemotherapy-induced vomiting in children: a meta-analysis
Source: Front Pharmacol. 2025 Jan 23;16:1509928. doi: 10.3389/fphar.2025.1509928 (PMC11798989; doi:10.3389/fphar.2025.1509928)
Supplement: Supplementary file 1 [file Supplementaryfile1.docx]

**Supplementary Material 1: Searching strategies**

**Pubmed**

#1 Chemotherapy induced nausea and vomiting [Title/Abstract] OR vomiting [Title/Abstract] OR Chemotherapy induced vomiting [Title/Abstract]

#2 fosaprepitant [Title/Abstract] OR antiemetic prophylaxis [Title/Abstract]

#3 pediatric patients [Title/Abstract] OR children [Title/Abstract] OR preschool [Title/Abstract]

#1 AND #2 AND #3

**Embase**

#1'Chemotherapy induced nausea and vomiting ' OR 'vomiting' OR ' Chemotherapy induced vomiting'

#2 'fosaprepitantl' OR 'antiemetic prophylaxis '

#3 ' pediatric patients' OR 'children ' OR 'preschool '

#1 AND #2 AND #3

**Cochrane Library**

#1( Chemotherapy induced nausea and vomiting):ti,ab,kw OR (vomiting):ti,ab,kw

#2 (fosaprepitant) :ti,ab,kw OR ( antiemetic prophylaxis ) :ti,ab,kw

#3 pediatric patients :ti,ab,kw OR children ti,ab,kw OR preschool :ti,ab,kw

#1 AND #2 AND #3
